# Comprehensive identification of ubiquitin-like 3 (UBL3)-interacting proteins in the mouse brain

**DOI:** 10.1186/s13041-024-01131-4

**Published:** 2024-08-15

**Authors:** Hiroshi Ageta, Tomoki Nishioka, Hisateru Yamaguchi, Kunihiro Tsuchida, Natsumi Ageta-Ishihara

**Affiliations:** 1 Division for Therapies Against Intractable Diseases, Center for Medical Science, Fujita Health University, 1-98 Dengakugakubo, Kutsukake-cho, Toyoake, Aichi 470-1192 Japan; 2 Open Facility Center, Research Promotion Headquarters, Fujita Health University, Toyoake, Aichi 470-1192 Japan; 3 Division of Cell Biology, International Center for Brain Science, Fujita Health University, Toyoake, Aichi 470-1192 Japan; 4 Department of Medical Technology, Yokkaichi Nursing and Medical Care University, Yokkaichi, 512-8045 Japan; 5 Department of Biomolecular Science, Faculty of Science, Toho University, 2-2-1 Miyama, Funabashi, Chiba 274-8510 Japan

**Keywords:** Post-translational modification, Ubiquitin-like 3 (UBL3), Proteomics, Small extracellular vesicles (sEVs), RNA binding proteins, Neurodegenerative diseases

## Abstract

Discovery of novel post-translational modifications provides new insights into changes in protein function, localization, and stability. They are also key elements in understanding disease mechanisms and developing therapeutic strategies. We have previously reported that ubiquitin-like 3 (UBL3) serves as a novel post-translational modifier that is highly expressed in the cerebral cortex and hippocampus, in addition to various other organs, and that 60% of proteins contained in small extracellular vesicles (sEVs), including exosomes, are influenced by UBL3. In this study, we generated transgenic mice expressing biotinylated UBL3 in the forebrain under control of the alpha-CaMKII promoter (*Ubl3*^Tg/+^). Western blot analysis revealed that the expression of UBL3 in the cerebral cortex and hippocampus was 6- to 7-fold higher than that in the cerebellum. Therefore, we performed immunoprecipitation of protein extracts from the cerebral cortex of *Ubl3*^+/+^ and *Ubl3*^Tg/+^ mice using avidin beads to comprehensively discover UBL3 interacting proteins, identifying 35 new UBL3 interacting proteins. Nine proteins were annotated as extracellular exosomes. Gene Ontology (GO) analysis suggested a new relationship between sEVs and RNA metabolism in neurodegenerative diseases. We confirmed the association of endogenous UBL3 with the RNA-binding proteins FUS and HPRT1—both listed in the Neurodegenerative Diseases Variation Database (NDDVD)—and with LYPLA1, which is involved in Huntington’s disease, using immunoprecipitation (IP)-western blotting analysis. These UBL3 interacting proteins will accelerate the continued elucidation of sEV research about proteins regulated by novel post-translational modifications by UBL3 in the brain.

## Main text

Post-translational modifications precisely regulate protein function and stability and play important roles in various cellular processes, including signal transduction [1, 2]. They are also very important in drug development because the precise control of target molecules involved in specific diseases and pathological conditions is an absolute requirement for designing effective therapies [3]. In the nervous system, post-translational modifications such as phosphorylation and ubiquitination are essential for the precise regulation of neurogenesis and synaptic function, and are also involved in the onset and progression of neurodegenerative diseases [4–6]. Therefore, posttranslational modifications play a crucial role in understanding disease mechanisms and developing therapeutic strategies.

We previously reported that ubiquitin-like 3 (UBL3)/membrane-anchored ubiquitin-fold protein is a novel post-translational modifier and that UBL3 regulates the transport of 60% of proteins contained in sEVs including exosomes [2, 7]. Furthermore, we comprehensively identified UBL3-associated molecules in MDA-MB-231 human breast cancer cells. Among these proteins, the oncogenic protein Ras undergoes UBL3 modification, is encapsulated in sEVs, is taken up by other cells, and activates growth signaling [7]. We have also reported that UBL3-interacting proteins identified in MDA-MB-231 cells include neurodegenerative disease-related proteins such as presenilin 1 and huntingtin-interacting protein 1-related protein. Although UBL3 is highly expressed in the brain [7], UBL3-associated molecules have not yet been identified in the brain. Therefore, we generated transgenic mice expressing UBL3 in the forebrain in this study and comprehensively identified UBL3-interacting proteins in the brain.

**Fig. 1:**
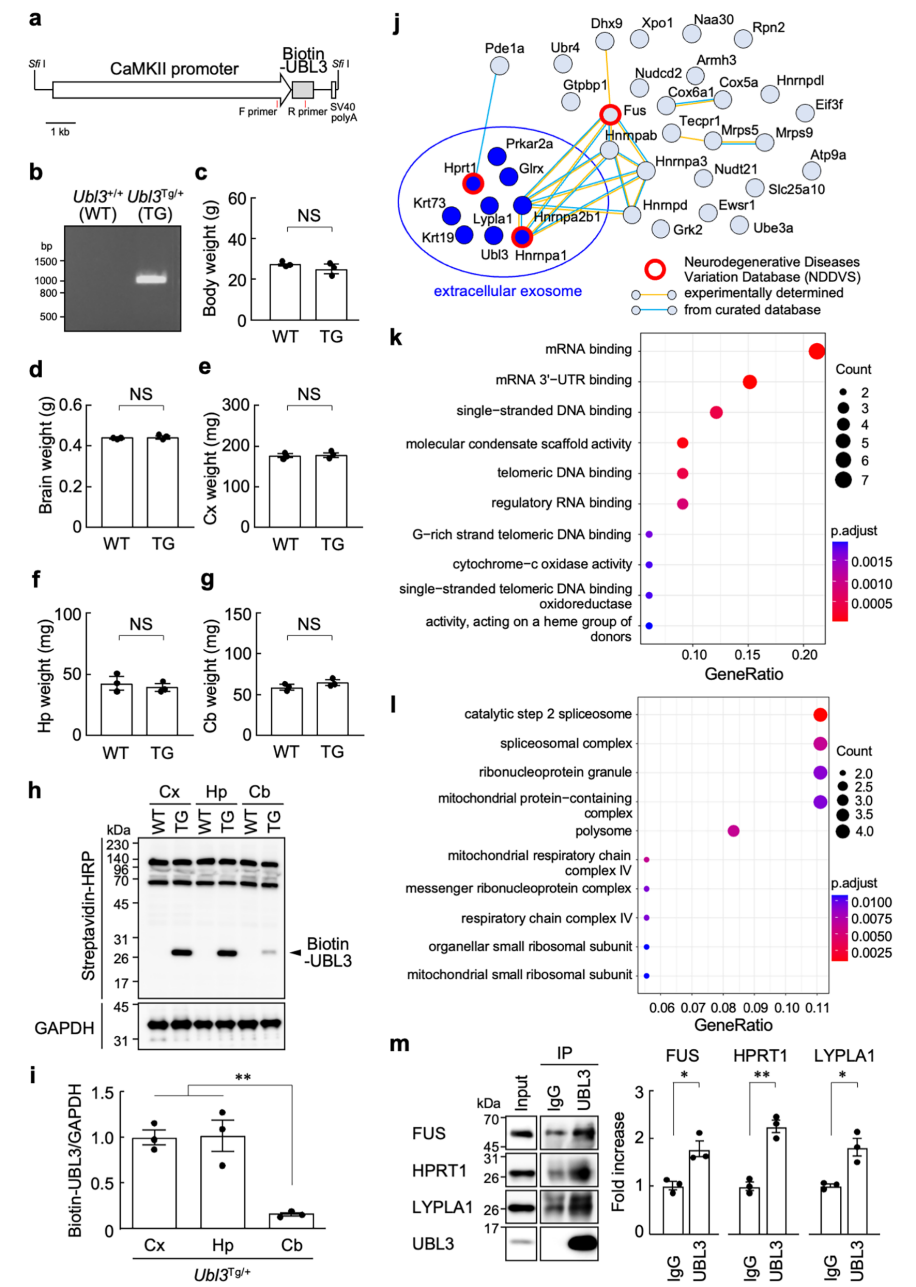
UBL3-interacting molecules identified from the cerebral cortex in transgenic mice overexpressing UBL3 under the control of the alpha-CaMKII promoter. **a**, Schematic representation of the UBL3 transgenic construct. Red lines indicate the location of the PCR primers used for genotyping. **b**, Representative agarose gel image of the RT-PCR genotyping. *Ubl3*^Tg/+^ mice showed an amplified transgene of *Ubl3* as 931 bp. Forward primer, 5’-CTTTCTCAAGGACCATCCCA-3’. Reverse primer, 5’-GCTGCTGACCTGCTCTTCTT-3’. **c**, Body weights of *Ubl3*^+/+^ and *Ubl3*^Tg/+^ male mice (9-10-week-old). **d**, Whole brain weights of *Ubl3*^+/+^ and *Ubl3*^Tg/+^ male mice (9-10-week-old). **e**, Cortex weight. **f**, Hippocampus weight. **g**, Cerebellum weight. **h**, **i**, Western blot analysis of the levels of biotinylated UBL3 and GAPDH (internal control) in proteins extracted from the cortex (Cx), hippocampus (Hp), and cerebellum (Cb) of *Ubl3*^+/+^ and *Ubl3*^Tg/+^ male mice (9-10-week-old) (**h**). Quantitative analysis of the normalized fraction of biotinylated UBL3 (**i**). **j**, Protein-protein interaction network of UBL3-interacting molecules in the mouse cortex obtained from the STRING database. The protein group surrounded by a blue circle was annotated as an extracellular exosome protein (GO:0070062). **k**, GO analysis of molecular functions of UBL3-interacting molecules. **l**, GO analysis for the cellular components of UBL3-interacting molecules. **m**, IP/western blot assay of UBL3 either with FUS, HPRT1, or LYPLA1 from *Ubl3*^+/+^ (9 week-old male mice) cerebral cortex lysates. Densitometric quantification of the relative amount of each protein. Data are mean ± s.e.m. **c**-**g**, **m**, Two-tailed unpaired *t* test. **i**, One-way ANOVA with Tukey’s multiple comparison test. *P < 0.05, **P < 0.01, NS, not significant

Because UBL3 mRNA has been detected in neurons in the Allen Brain Atlas, we constructed a transcription unit by inserting an artificially synthesized coding region for mouse *Ubl3* with a biotinylated tag added to the N-terminus into the alpha-CaMKII promoter. *Sfi* I fragments (Fig. 1a) were microinjected into the pronuclei of one-cell embryos of C57BL/6J mice to produce transgenic mice using previously reported methods [8]. Microinjected embryos were transferred to the oviducts of pseudo-pregnant females. Founder transgenic mice were identified by PCR using genomic DNA extracted from their tails (Fig. 1b) and bred with C57BL/6J mice (CLEA). Between *Ubl3*^+/+^ (wild type; WT) and *Ubl3*^Tg/+^ (transgenic; TG) male mice (9-10-week-old), no differences were observed for body (Fig. 1c) or brain weight (Fig. 1d). As we previously confirmed endogenous UBL3 expression in the cerebral cortex, hippocampus, and cerebellum using western blotting [7], we measured the respective weights of these tissues in *Ubl3*^+/+^ and *Ubl3*^Tg/+^ male mice. We did not detect statistically significant differences in the weight of the cortex (Fig. 1e), hippocampus (Fig. 1f), or cerebellum (Fig. 1g). Therefore, we quantified the amount of biotinylated UBL3 overexpressed in the cerebral cortex, hippocampus, and cerebellum. The amount of overexpressed UBL3 detected with a streptavidin-HRP (Invitrogen, 19534-050, 1:5000) was normalized to the amount of endogenous control protein detected with a glyceraldehyde 3-phosphate dehydrogenase (GAPDH) antibody (Cell Signaling, 2118, 1:1000). The results showed that UBL3 expression was significantly higher in the cerebral cortex and hippocampus than in the cerebellum (Fig. 1h, i). Because sufficient protein content was extracted from the cortex compared with the hippocampus, each protein extract from the cortex of *Ubl3*^+/+^ and *Ubl3*^Tg/+^ male mice (9-10-week-old, *n* = 3 in each group) was incubated with 30 µL of NeutrAvidin Agarose (ThermoFisher, 29202) for 18 h at 4 °C and subjected the proteins to mass spectrometry using previously reported methods [7]. The peptides were analyzed by liquid chromatography/mass spectrometry (LC/MS) using an Orbitrap Fusion mass spectrometer coupled to an Easy-nLC 1000 using an EASY-Spray ES900 column system (ThermoFisher, 75 μm × 150 mm, particle size 3 μm). Mass spectrometry raw files were processed using Proteome Discoverer version 2.4 (ThermoFisher) and a local MASCOT server (version 2.6.2; Matrix Science). The MS/MS data were searched against *Mus musculus* (SwissProt TaxID = 10090_and_subtaxonomies) (v2017-10-25). We identified 35 UBL3 interacting molecules with an increase of 1.5 times or more in the *Ubl3*^Tg/+^ mice compared to the *Ubl3*^+/+^ mice and an experimental q-value of 0.05 or less (Table 1). When a protein-protein interaction network was created using the STRING database, 9 out of 35 UBL3-binding molecules were annotated as extracellular exosome (GO:0070062) (Fig. 1j). The percentage of molecules annotated as exosomes was 25%, which was similar to the comprehensive proteomic analysis of UBL3 interacting proteins conducted in breast cancer cells [7]. The ClusterProfiler R package was used for GO enrichment analysis of these UBL3 interacting proteins, which categorized these molecules as RNA binding proteins (Fig. 1k, l). Recent studies have focused on the relationship between abnormalities in RNA metabolism caused by the disruption of RNA binding proteins and neurodegenerative diseases [9]. Interestingly, the UBL3 interacting proteins identified in this study included the RNA binding proteins FUS, Hnrnpa1, and Hprt1, which are included in the 289 genes registered in the Neurodegenerative Diseases Variation Database (NDDVD, http://www.sysbio.org.cn/NDDVD/diseases). Protein extract from the cortex of *Ubl3*^+/+^ male mice (9-week-old, *n* = 3 in each group) were incubated with 25 µL of Protein G Sepharose (Cytiva, 17-0618-01) and 2 µg of normal rabbit immunoglobulin G (IgG) (Cell Signaling, 2729 S) or anti-UBL3 antibody (Proteintech, 14100-1-AP, lot 00005139) for 18 h at 4 °C and subjected western blotting either with anti-FUS (Santa Cruz, sc-47711, 1:200) or anti- HPRT1 (abcam, ab109021, 1:20000) antibodies. As a result, we showed that endogenous UBL3 is associated with FUS and HPRT1 (Fig. 1m). TDP-43, which is related to neurodegenerative diseases, has been reported to be encapsulated in sEVs [10] and binds to FUS [11]. Whether the transport of TDP-43 to sEVs is mediated by the UBL3 modification of FUS is a subject for future studies. If the relationship between RNA metabolism and protein transport to sEVs in neurodegenerative diseases is elucidated, compounds that affect UBL3 modification may become novel drug candidates, employing innovative therapeutic strategies for neurodegenerative diseases involving RNA metabolism. We previously reported that huntingtin-interacting protein 1-related protein associates with UBL3 in MDA-MB-231 cells [7]. Interestingly, among the UBL3 interacting molecules in the cerebral cortex, we identified Lypla1/APT1, a molecule involved in the pathogenesis of Huntington’s disease [12], and confirmed its binding to UBL3 using immunoprecipitation (IP)-western blotting with anti-LYPLA1 antibody (Proteintech, 16055-1-AP, 1:2000) (Fig. 1m). This result suggests that UBL3 could be involved in the pathogenesis and/or progression of neurodegenerative diseases.

In this study, we successfully established transgenic mice that highly expressed biotinylated UBL3, specifically in the forebrain. Beta-amyloid in Alzheimer’s disease and alpha-synuclein in Parkinson’s disease are known to be sorted through sEVs [13, 14]. We have previously reported that 60% of proteins sorted into sEVs are UBL3-dependent [7]. sEVs research in the field of neuroscience is expected to be accelerated by crossbreeding mouse models of sEV-associated neurodegenerative diseases with the newly established mice overexpressing UBL3 in the forebrain.

**Table 1:** The list of 35 UBL3 interacting proteins

	Accession No.	Description	# AAs	Gene Symbol	extracellular exosome	Neurodegenerative Diseases Variation Database
1	P12787	Cytochrome c oxidase subunit 5 A, mitochondrial [OS=Mus musculus]	146	Cox5a	-	-
2	Q61545	RNA-binding protein EWS [OS=Mus musculus]	655	Ewsr1	-	-
3	P56959	RNA-binding protein FUS [OS=Mus musculus]	518	Fus	-	+
4	Q99MK8	Beta-adrenergic receptor kinase 1 [OS=Mus musculus]	689	Grk2	-	-
5	O08582	GTP-binding protein 1 [OS=Mus musculus]	668	Gtpbp1	-	-
6	P19001	Keratin, type I cytoskeletal 19 [OS=Mus musculus]	403	Krt19	+	-
7	Q6NXH9	Keratin, type II cytoskeletal 73 [OS=Mus musculus]	539	Krt73	+	-
8	P97823-1	Acyl-protein thioesterase 1 [OS=Mus musculus]	230	Lypla1	+	-
9	Q9D7N3	28 S ribosomal protein S9, mitochondrial [OS=Mus musculus]	390	Mrps9	-	-
10	Q9Z2M6	Ubiquitin-like protein 3 [OS=Mus musculus]	117	Ubl3	+	-
11	A2AN08	e3 ubiquitin-protein ligase UBR4 [OS=Mus musculus]	5180	Ubr4	-	-
12	Q99020	Heterogeneous nuclear ribonucleoprotein A/B [OS=Mus musculus]	285	Hnrnpab	-	-
13	Q9Z130	Heterogeneous nuclear ribonucleoprotein D-like [OS=Mus musculus]	301	Hnrnpdl	-	-
14	Q6PD19-1	UPF0668 protein C10orf76 homolog [OS=Mus musculus]	689	Armh3	-	-
15	O70133-2	Isoform 2 of ATP-dependent RNA helicase A [OS=Mus musculus]	1381	Dhx9	-	-
16	Q9QZD8	Mitochondrial dicarboxylate carrier [OS=Mus musculus]	287	Slc25a10	-	-
17	P49312-1	Heterogeneous nuclear ribonucleoprotein A1 [OS=Mus musculus]	320	Hnrnpa1	+	+
18	Q99N87	28 S ribosomal protein S5, mitochondrial [OS=Mus musculus]	432	Mrps5	-	-
19	O70228	Probable phospholipid-transporting ATPase IIA [OS=Mus musculus]	1047	Atp9a	-	-
20	P00493	Hypoxanthine-guanine phosphoribosyltransferase [OS=Mus musculus]	218	Hprt1	+	+
21	Q9QUH0	Glutaredoxin-1 [OS=Mus musculus]	107	Glrx	+	-
22	O88569	heterogeneous nuclear ribonucleoproteins A2/B1 [OS=Mus musculus]	353	Hnrnpa2b1	+	-
23	Q9DCH4	Eukaryotic translation initiation factor 3 subunit F [OS=Mus musculus]	361	Eif3f	-	-
24	Q61481	Dual specificity calcium/calmodulin-dependent 3’,5’-cyclic nucleotide phosphodiesterase 1 A OS=Mus musculus OX=10,090 GN=Pde1a PE=1 SV=3	545	Pde1a	-	-
25	Q8CES0-1	N-alpha-acetyltransferase 30 [OS=Mus musculus]	364	Naa30	-	-
26	P43024	Cytochrome c oxidase subunit 6A1, mitochondrial [OS=Mus musculus]	111	Cox6a1	-	-
27	Q9DBG6	Dolichyl-diphosphooligosaccharide--protein glycosyltransferase subunit 2 [OS=Mus musculus]	631	Rpn2	-	-
28	Q9CQ48	NudC domain-containing protein 2 [OS=Mus musculus]	157	Nudcd2	-	-
29	P12367	cAMP-dependent protein kinase type II-alpha regulatory subunit [OS=Mus musculus]	401	Prkar2a	+	-
30	Q9CQF3	Cleavage and polyadenylation specificity factor subunit 5 [OS=Mus musculus]	227	Nudt21	-	-
31	O08759	Ubiquitin-protein ligase E3A [OS=Mus musculus]	870	Ube3a	-	-
32	Q80VP0-1	Tectonin beta-propeller repeat-containing protein 1 [OS=Mus musculus]	1166	Tecpr1	-	-
33	Q6P5F9	Exportin-1 [OS=Mus musculus]	1071	Xpo1	-	-
34	Q60668-1	heterogeneous nuclear ribonucleoprotein D0 [OS=Mus musculus]	355	Hnrnpd	-	-
35	Q8BG05	Heterogeneous nuclear ribonucleoprotein A3 [OS=Mus musculus]	379	Hnrnpa3	-	-

## Data Availability

All data analyzed during this study are included in this published article and its additional file. Proteomics raw datasets are deposited in jPOST (under accession codes JPST002396, PXD047087).

## Abbreviations

UBL3: Ubiquitin-like 3
sEVs: Small extracellular vesicles
GO: Gene Ontology
WT: Wild type
TG: Transgenic
GAPDH: Glyceraldehyde 3-phosphate dehydrogenase
NDDVD: Neurodegenerative Diseases Variation Database
IgG: Immunoglobulin G
IP: Immunoprecipitation
